# Comparison of plantar pressure distribution, balance and functional parameters in older adults with high and low fall risk: a cross-sectional study

**DOI:** 10.1007/s11845-026-04286-2

**Published:** 2026-02-24

**Authors:** Hüseyin Gerdan, Gözde Kesikbaş Kurt, Gonca Üstünbaş Atmaca, Kamil Yılmaz, Nilgün Bek

**Affiliations:** 1 Vocational School of Health Services, Department of Medical Services and Techniques, Ondokuz Mayıs University, Samsun, Turkey; 2 Faculty of Health Sciences, Department of Orthosis and Prosthesis, Ondokuz Mayıs University, Samsun, Turkey; 3 Atakum Nursing Home, Elderly Care and Rehabilitation Center, Samsun, Turkey; 4 Faculty of Health Sciences, Department of Physiotherapy and Rehabilitation, Lokman Hekim University, Ankara, Turkey

**Keywords:** Older adults, Fall risk, Plantar pressure, Balance, Gait

## Abstract

**Background:**

Falls are a common and serious problem among older adults, often leading to injury and functional decline. Understanding plantar pressure patterns and functional performance can help identify fall risk mechanisms.

**Aims:**

This study aimed to compare plantar pressure distribution, balance, and functional parameters in older adults with high and low fall risk.

**Methods:**

Twenty-nine older adults (mean age 72.76 ± 5.23 years) participated. Participants were divided based on Berg Balance Scale scores: high fall risk (*n* = 15) and low fall risk (*n* = 14). Static and dynamic plantar pressures were assessed using pedobarography. Functional mobility and balance were evaluated with the Functional Reach Test (FRT), Timed Up and Go Test (TUG), and 10-Meter Walk Test (10MWT).

**Results:**

No significant differences were observed in static plantar pressure parameters. In dynamic analysis, non-dominant forefoot impulse was significantly higher in the low fall-risk group (*p* = 0.032), while dominant midfoot impulse was lower in the low fall-risk group (*p* = 0.041). Low-risk participants reached longer distances in FRT (*p* = 0.001) and completed TUG faster (*p* = 0.010), indicating better functional performance. 10MWT performance was better in the low-risk group but not statistically significant (*p* = 0.085).

**Conclusions:**

Static plantar pressure parameters did not differ significantly between groups, but dynamic plantar pressure and functional tests did. Better FRT and TUG performance and higher non-dominant forefoot impulse were observed in the low-risk group. Evaluating fall risk should incorporate balance, time-dependent plantar pressure, and functional performance, guiding intervention strategies.

**Supplementary Information:**

The online version contains supplementary material available at 10.1007/s11845-026-04286-2.

## Background

Age-related declines in muscle strength, proprioception, and sensory feedback contribute to impairments in postural control and increase the risk of falls among older adults [1, 2]. These changes often lead to serious injuries and a loss of independence [3]. Falls are a major public health concern worldwide, as fall-related injuries and hospitalizations continue to rise. Fractures, head trauma, and other fall-induced injuries not only reduce quality of life but also impose a significant burden on healthcare systems [4, 5].

Biomechanical alterations associated with aging, such as reduced joint stability, changes in plantar loading patterns, and altered gait mechanics negatively affect balance and mobility in older adults [6]. The quantitative assessment of these biomechanical parameters through force or pressure platforms provides objective insights into postural control mechanisms [7]. In particular, plantar pressure and center of pressure (CoP) parameters are closely related to balance and gait stability in older adults [8]. Evaluating both static (standing balance) and dynamic (during gait or functional movement) parameters has been shown to be useful for identifying individuals at higher fall risk [9, 10].

Reduced plantar sensory input further disrupts pressure distribution under the foot, compromising postural stability [11]. Beyond postural analysis, plantar pressure assessment offers detailed information about changes in foot loading patterns that may reflect compensatory balance strategies. Foot biomechanics and plantar pressure asymmetries are significant contributors to fall risk, emphasizing the importance of dynamic foot loading assessment. Increased loading in the forefoot and heel regions has been shown to alter postural stability and may contribute to a higher risk of falls in older adults [9, 12]. Although previous studies have mostly focused on general fall frequency or risk classification [13, 14], few have simultaneously examined static and dynamic plantar pressure parameters together with functional mobility measures such as gait speed and functional reach tests. Unlike previous research, this study concurrently examined static and dynamic plantar pressure parameters together with functional mobility tests, providing a more comprehensive understanding of fall risk mechanisms in older adults.

Therefore, this cross-sectional study aimed to compare plantar pressure distribution, balance, and functional mobility parameters between older adults with high and low fall risk. We hypothesized that individuals with higher fall risk would exhibit altered plantar pressure and CoP parameters, along with poorer balance and functional performance outcomes.

## Methods

### Participants

This cross-sectional study included older adults referred to our clinic for plantar pressure distribution analysis between April and July 2025. All participants provided written informed consent before data collection. The study was approved by the XXXXXXXXX Clinical Research Ethics Committee (Date: March 3, 2025; Approval No: 2025/113).

Inclusion criteria were: age ≥ 65 years, ability to walk independently for at least 10 m, and a Standardized Mini-Mental Test (SMMT) score ≥ 24 [15]. Exclusion criteria included inability to walk without assistance, severe musculoskeletal or neurological disorders, orthostatic hypotension, or cognitive impairments affecting comprehension of test instructions.

**Fig. 1:**
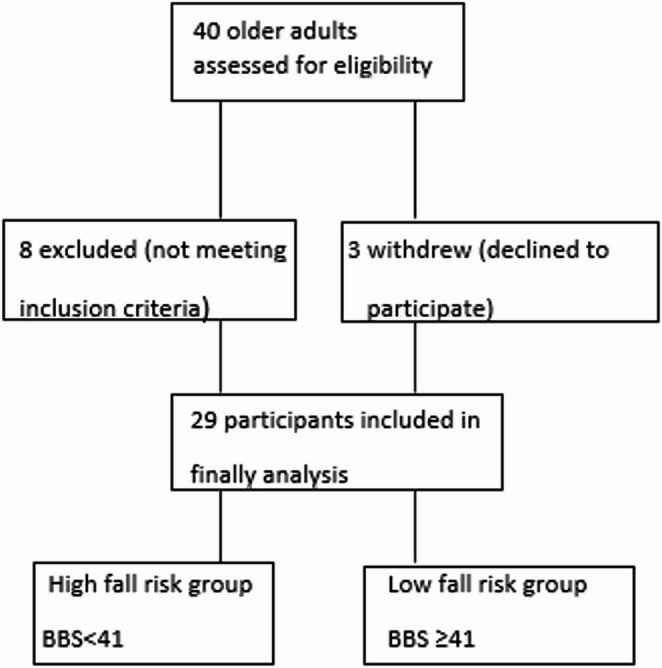
Flow diagram of participant recruitment and grouping

A total of 40 older adults were initially assessed for eligibility. Eight individuals did not meet the inclusion criteria and were therefore excluded. Additionally, three participants withdrew from the study before completion. As a result, 29 participants met the eligibility criteria and were included in the final analysis. In accordance with Şahin et al. (2013), a BBS cutoff score of 41 points was used to classify participants [13]. Based on the Berg Balance Scale (BBS) scores, participants were categorized into two groups: a high fall risk group (BBS < 41, *n* = 15) and a low fall risk group (BBS ≥ 41, *n* = 14) (Fig. 1). Comparisons were made between these two groups.

A priori power analysis was conducted using G*Power 3.1 software. Based on the parameters reported by Ünver et al. (2014) (effect size = 0.5, α = 0.05, power = 0.95), a minimum of 13 participants per group was required [14].

Demographic characteristics including age, height, weight, and dominant lower extremity were recorded. The dominant leg was determined by asking, “Which foot would you use to kick a ball?” [16]. Height and weight were measured barefoot using a stadiometer and calibrated digital scale, respectively, and Body Mass Index (BMI) was calculated as weight (kg) / height² (m²).

### Plantar pressure and center of pressure (CoP) assessment

Plantar pressure distribution was measured using a pressure platform (Analiz Sistem^®^, Turkey; 400 × 400 mm, 2288 sensors) with established validity and reliability [17]. *Static analysis*: Participants stood barefoot with heels 5 cm apart and feet at a 30° angle to the frontal plane, arms relaxed by their sides, and eyes fixed forward. Each trial lasted 10 s. Parameters recorded included maximum and mean pressure, contact area per foot, and pressure distribution (%) in forefoot and hindfoot regions. CoP sway along the mediolateral (X) and anteroposterior (Y) axes was automatically calculated in millimeters to quantify postural stability. *Dynamic analysis*: Participants walked barefoot at a self-selected pace along a 3-meter walkway incorporating the pressure platform. During the stance phase, the following parameters were obtained for forefoot, midfoot, and rearfoot regions: pressure percentage, impulse, and contact surface area. *Impulse* was defined as the time integral of vertical ground reaction force (force×time), expressed in Newton-seconds (N·s), reflecting both the magnitude and duration of foot-ground contact. *Surface area* (mm²) represented the contact area of each foot region during stance. Together, these parameters provide objective information about load distribution and gait mechanics.

### Functional balance and mobility tests

Functional mobility and fall risk were further evaluated using validated clinical tests.

- Timed Up and Go (TUG): Participants rose from a 46 cm chair, walked 3 m, turned around, returned, and sat down. Completion time (s) was recorded [18].
- 10-Meter Walk Test (10MWT): Participants walked 10 m at a comfortable pace; walking time was recorded and used to calculate gait speed [19].
- Functional Reach Test (FRT): Standing with feet shoulder-width apart, participants reached forward as far as possible without stepping or losing balance. The mean of three attempts (cm) after one practice trial was used for analysis [20].

SMMT scores (for eligibility) and BBS scores (for fall risk classification) were also recorded.

### Statistical analysis

Data were analyzed using SPSS version 19.0 (IBM Corp., Armonk, NY, USA). The Shapiro–Wilk test was used to assess normality. For normally distributed variables, independent-samples *t*-tests were used; otherwise, the Mann–Whitney U test was applied. Categorical variables were compared using Chi-square (χ²) tests. Statistical significance was set at *p* < 0.05.

Based on the preliminary power analysis, the achieved effect size was 1.535, and the statistical power (1–β) was 0.95, confirming that a minimum of 13 participants per group was sufficient.

## Results

A total of 29 older adults, including 13 females and 16 males, with a mean age of 72.76 ± 5.23 years, participated in the study. A significant difference was found between the groups in terms of Berg Balance Scale scores used for group classification. However, no statistically significant differences were found between the groups regarding age, gender distribution, Body Mass Index (BMI), or Standardized Mini Mental Test (SMMT) scores (*p* > 0.05) (Table 1).

**Table 1:** Demographic characteristics of the participants and comparison between groups

		High Fall Risk Group (*n* = 15)		Low Fall Risk Group (*n* = 14)		*p*
		Min-Max	Mean ± SD	Min-Max	Mean ± SD	
Age		65–82	73.80 ± 5.20	65–84	71.64 ± 5.23	0.275^a^
BMI (kg/m^2^)		20.82–39.06	29.45 ± 6.05	15.94–39.54	27.05 ± 5.66	0.282^a^
BBS		26–40	33.53 ± 4.49	41–50	44.57 ± 2.41	< 0.001*^,a^
SMMT		25–28	26.80 ± 1.08	26–29	26.57 ± 1.16	0.587^a^
Gender	Male n (%)	8 (53.3)		5 (35.7)		0.340^b^
	Female n (%)	7 (46.7)		9 (64.3)		

*p < 0.05: Statistical significance; n: Number of participants; Min: Minimum; Max: Maximum; Mean: Average; SD: Standard deviation; BMI: Body Mass Index; SMMT: Standardized Mini-Mental Test Score; a: Independent Samples t-test; b: Pearson Chi-Square test

The arithmetic mean and standard deviation values of all measured parameters, along with the statistical comparisons and differences between groups, are presented in Table 2 (Table 2).

**Table 2:** Comparison of the groups in terms of plantar pressure distribution, balance, and functional parameters

			High Fall Risk Group (*n* = 15)	Low Fall Risk Group(*n* = 14)	t or z	p	ES
			X ± SD	X ± SD			
Static plantar pressure distribution	**Foot** **Pressure (g/cm** ^**2**^ **)**	**D**	50.19 ± 2.85	52.51 ± 8.92	-0.497	0.747^a^	
		**N-D**	49.81 ± 2.85	47.49 ± 8.92	-0.497	0.747^a^	
	**Forefoot pressure (g/cm** ^**2**^ **)**	**D**	26.79 ± 5.80	29.03 ± 3.95	-1.207	0.238^b^	
		**N-D**	26.42 ± 5.96	26.49 ± 5.67	-0.029	0.977^b^	
	**Rearfoot pressure (g/cm** ^**2**^ **)**	**D**	23.42 ± 5.82	23.86 ± 8.19	-0.044	0.983^a^	
		**N-D**	23.37 ± 6.11	20.99 ± 5.78	1.077	0.291^b^	
	**Surface (mm** ^**2**^ **)**	**D**	213.88 ± 47.00	216.67 ± 11.40	-0.167	0.868^b^	
		**N-D**	213.69 ± 46.98	215.95 ± 40.31	-0.139	0.890^b^	
Dynamic plantar pressure distribution	**Forefoot surface (mm** ^**2**^ **)**	**D**	37.45 ± 9.30	40.92 ± 9.12	-1.015	0.319^b^	
		**N-D**	38.29 ± 7.13	40.54 ± 6.97	-1.179	0.252^a^	
	**Midfoot surface (mm** ^**2**^ **)**	**D**	28.35 ± 10.22	23.45 ± 8.42	1.402	0.172^b^	
		**N-D**	25.76 ± 6.29	25.33 ± 6.53	0.181	0.858^b^	
	**Rearfoot surface (mm** ^**2**^ **)**	**D**	34.21 ± 10.80	35.62 ± 7.66	-0.402	0.691^b^	
		**N-D**	35.95 ± 7.13	33.49 ± 6.24	0.989	0.332^b^	
	**Forefoot impulse (N.s)**	**D**	33.32 ± 10.10	39.11 ± 14.11	-1.277	0.212^b^	
		**N-D**	36.91 ± 13.23	47.20 ± 11.18	-2.255	0.032* ^,**b**^	**d = 0.87**
	**Midfoot impulse (N.s)**	**D**	31.45 ± 12.51	22.14 ± 8.03	-2.052	0.041* ^,**a**^	*r* = 0.38
		**N-D**	24.47 ± 10.62	17.76 ± 8.37	-1.812	0.070^a^	
	**Rearfoot impulse (N.s)**	**D**	35.36 ± 10.93	38.76 ± 9.19	-0.902	0.375^b^	
		**N-D**	38.63 ± 10.20	35.04 ± 9.34	0.986	0.333^b^	
CoP	**Deviation from the X-axis**		27.57 ± 14.59	21.58 ± 17.00	1.020	0.317^b^	
	**Deviation from the Y-axis**		37.06 ± 19.99	24.81 ± 18.43	1.711	0.098^b^	
Functional tests	**10 m gait test (sec.)**		13.52 ± 2.48	11.83 ± 2.43	-1.746	0.085^a^	
	**FRT (cm)**		23.97 ± 3.37	28.68 ± 3.74	-3.565	0.001* ^,**b**^	**d = 1.37**
	**TUG (sec.)**		15.58 ± 3.37	12.58 ± 2.35	2.764	0.010* ^,**b**^	**d = 1.06**

*p < 0.05 indicates statistical significance. D dominant, N-D non-dominant, CoP, center of pressure, FRT Functional Reach Test, TUG Timed Up and Go. Test statistic: t for independent-samples t-test, z for Mann–Whitney U test. a: independent-samples t-test; b: Mann–Whitney U test. ES: effect size (Cohen’s d for t-test; r = Z/√N for Mann–Whitney U)*

No significant difference was found between the two groups in terms of static plantar pressure distribution and CoP deviation values (*p* > 0.05). However, when the dynamic plantar pressure distribution data were examined, the forefoot impulse value of the non-dominant foot was found to be higher in the group with a lower risk of falling (*p* = 0.032), while the midfoot impulse value of the dominant foot was found to be lower (*p* = 0.041).

When the results of the functional mobility and balance tests were examined, the group with a lower risk of falling appeared to have better performance in the 10-meter walk test; however, this difference was not statistically significant. On the other hand, individuals in the low fall risk group reached a longer distance in the FRT and completed the TUG test in a shorter time, both of which were statistically significant (*p* < 0.05).

## Discussion

Balance loss and falls in older adults are major public health concerns, as they reduce quality of life and threaten individual independence [21]. Age-related proprioceptive decline, reduced muscle strength, and sensory changes in the plantar surface have been shown to contribute to increased fall risk [22]. In particular, the distribution of plantar pressure is a critical parameter for both postural control and gait stability [23]. Therefore, this study was conducted to compare plantar pressure distribution, balance, and functional parameters in older adults with different levels of fall risk. The findings revealed that individuals with a higher risk of falling showed negative alterations particularly in dynamic plantar pressure parameters, as well as in assessments such as the FRT and TUG test.

No significant difference was found between the groups in terms of static plantar pressure distribution. This finding suggests that static pressure measurements may have limited value in distinguishing fall risk and that postural stability in older adults tends to deteriorate more prominently during dynamic processes. Indeed, it is well known that impaired postural control in geriatric individuals typically becomes more evident during dynamic activities such as walking, turning, or obstacle negotiation [24]. In our study, when the dynamic plantar pressure data were analyzed, the impulse value in the non-dominant forefoot was found to be significantly higher in the low fall risk group. This may indicate that effective force transmission and muscular control in the forefoot play a role in reducing fall risk. Furthermore, the significantly lower impulse in the dominant midfoot observed in the low-risk group suggests that directing load transfer toward more distal areas of the foot may enhance the contribution of the forefoot and toe muscles to balance, thereby providing an advantage in terms of postural stability. A lower midfoot impulse in the low fall-risk group may reflect more efficient load transfer and better activation of distal foot muscles, contributing to improved balance control [25]. These findings highlight the potential of dynamic plantar pressure distribution to serve as a determinant of fall risk and are consistent with previous studies that emphasize the relationship between foot biomechanics and fall risk [26, 27].

No significant difference was observed between the groups in terms of CoP parameters. This finding suggests that CoP measurements obtained under static conditions and during short-term assessments may have limited predictive value for fall risk. In the literature, it has been reported that CoP variability gains more significance particularly in long-duration dynamic analyses [28]. A meta-analysis conducted in 2020 emphasized that commonly used clinical tests for predicting fall risk in older adults lack sufficient sensitivity and highlighted the need for more objective methods to assess postural stability. Although CoP measurements are among the most frequently used laboratory-based assessments for evaluating postural control, there is still no consensus on which specific features of the CoP trajectory can serve as reliable biomarkers for fall risk [29]. Clinically, increased mediolateral CoP deviations may indicate reduced lateral stability, whereas anteroposterior fluctuations may represent compensatory postural strategies. Therefore, dynamic CoP analysis can provide valuable insights into postural control mechanisms that are not captured through static assessments [28, 29].

In functional mobility and balance tests, the significantly better performance of the low fall risk group indicates the effectiveness of these tests in distinguishing fall risk. Specifically, longer reach distances in the Functional Reach Test and shorter times in the Timed Up and Go test reflect the relationship between fall risk and postural control [30, 31]. However, although the low-risk group performed better in the 10-meter walk test, this difference was not statistically significant. This suggests that gait speed alone has limited accuracy in predicting fall risk. As highlighted in the systematic review by Beck Jepsen et al. (2022) [32], no single balance, gait, or functional mobility test is sufficient to predict fall risk in older adults, while gait speed may be useful as part of a multidimensional assessment. Consistent with the literature, we believe that a comprehensive and multidimensional evaluation provides a more accurate assessment of fall risk.

Based on these findings, we suggest that dynamic plantar pressure patterns and functional performance parameters may provide valuable guidance for developing rehabilitation strategies aimed at improving balance and reducing fall risk in older adults.

Clinically, these findings suggest that fall-risk assessment and rehabilitation in older adults should integrate dynamic plantar pressure patterns with functional performance measures, rather than relying solely on static evaluations. Interventions targeting distal foot control, such as foot–toe and intrinsic foot muscle strengthening, together with sensorimotor and proprioceptive training, may help optimise load transfer and dynamic stability [9, 29]. Future intervention studies are needed to determine whether such targeted approaches can modify dynamic plantar pressure patterns and reduce fall incidence.

### Limitations

One limitation of this study is that fall risk was assessed solely using the Berg Balance Scale, without the inclusion of a specific fall-related instrument such as the Falls Efficacy Scale, which may have limited the comprehensive evaluation of fall risk. Additionally, the dynamic plantar pressure distribution analysis was conducted using a relatively small sensor-embedded platform integrated into the walking pathway, which may have influenced the measurements. Due to the cross-sectional design, causal relationships between plantar pressure parameters and actual fall events cannot be established; therefore, prospective longitudinal studies are required to determine whether dynamic pressure changes predict future falls. Furthermore, the relatively small sample size (*n* = 29) limits the generalisability of the findings. The lack of systematic consideration of comorbidities (e.g., diabetes mellitus and cardiovascular disease) represents a limitation of the study, as these conditions may influence plantar pressure characteristics and functional outcomes. Similarly, the absence of direct assessment of peripheral muscle strength constitutes a limitation, given the well-established role of muscle strength in balance control and fall risk.

## Conclusions

This study aimed to compare dynamic and static plantar pressure distribution, balance, and functional mobility parameters between older adults with high and low fall risk, investigating how changes in these parameters affect increased fall risk. While no significant differences were found in static plantar pressure parameters, changes in dynamic pressure distribution were identified as potential contributors to increased fall risk. Functional mobility and balance tests effectively distinguished individuals with low fall risk, indicating their importance as clinical assessment tools. However, gait speed alone was insufficient to predict fall risk. These findings underscore the necessity of multidimensional and comprehensive evaluations in determining fall risk among older adults. Future studies with larger sample sizes and prospective follow-up are recommended to explore these relationships in greater detail.

## Supplementary Information

Below is the link to the electronic supplementary material.

Supplementary Material 1 (DOCX 182 KB)

Supplementary Material 2 (DOCX 180 KB)

## Data Availability

The datasets used and/or analysed the current study are available from the corresponding author on reasonable request.
